# Novice and Advanced Learners’ Satisfaction and Perceptions of an e-Learning Renal Semiology Module During the COVID-19 Pandemic: Mixed Methods Study

**DOI:** 10.2196/29216

**Published:** 2021-06-28

**Authors:** Ido Zamberg, Eduardo Schiffer, Catherine Stoermann-Chopard

**Affiliations:** 1 Division of Anesthesiology Department of Anesthesiology, Emergency Medicine, Clinical Pharmacology and Intensive Care Geneva University Hospitals Geneva Switzerland; 2 School of Education Johns Hopkins University Baltimore, MD United States; 3 Unit of Development and Research in Medical Education University of Geneva Geneva Switzerland; 4 Division of Nephrology Department of Medicine Geneva University Hospitals Geneva Switzerland

**Keywords:** COVID-19, e-learning, medical education, eHealth, novice, advanced, students, undergraduate education, satisfaction, perception, renal, qualitative, prospective, case study, design, clinical skills, clinical skills education

## Abstract

**Background:**

Nephrotic syndrome is a unique clinical disorder, which provides interesting teaching opportunities that connect physiological and pathological aspects to clinical practice. During the current COVID-19 outbreak, in-person teaching in our institution was not permitted, thus creating a unique challenge for clinical skills teaching. A case-based electronic learning (e-learning) activity was designed to replace the traditional in-person teaching of renal semiology. e-Learning activities have been shown to be effective for knowledge retention and increasing novice learners’ performance. However, major knowledge gaps exist concerning the satisfaction of learners with e-learning activities as the sole form of teaching, specifically for undergraduate clinical skills education.

**Objective:**

Our study aimed to prospectively assess undergraduate medical students’ perceptions of and satisfaction with an e-learning activity teaching renal semiology.

**Methods:**

All second-year medical students (novice learners) from the medical faculty of the University of Geneva, Switzerland, undertook the e-learning activity and were invited to participate in a nonmandatory, validated web-based survey, comprising questions answered using a 10-point Likert scale and one qualitative open-ended question. For comparison and to provide further insights, 17 fourth- to sixth-year students (advanced learners) were prospectively recruited to participate in both the e-learning activity and the evaluation. A mixed methods analysis was performed.

**Results:**

A total of 88 (63%) out of 141 novice learners and all advanced learners responded to the evaluation survey. Advanced learners reported significantly higher satisfaction with the e-learning activity (mean 8.7, SD 1.0 vs mean 7.3, SD 1.8; *P*<.001), clarity of objectives (mean 9.6, SD 0.8 vs mean 7.7, SD 1.7; *P*<.001), and attainability of objectives (mean 9.8, SD 0.5 vs mean 7.3, SD 1.3; *P*<.001). Both groups showed high interest in the inclusion of the activity as part of a blended learning approach; however, there was low interest in the activity being the sole means of teaching.

**Conclusions:**

Case-based e-learning activities might be better suited for advanced learners and could increase learners’ satisfaction within a blended teaching instructional design. More research on students’ satisfaction with e-learning activities in the field of clinical skills education should be done. In addition, more effort should be put into finding alternative teaching tools for clinical skills education in light of the ongoing COVID-19 pandemic and future health crises.

## Introduction

Nephrotic syndrome, a potentially life-threatening clinical disorder, occurs due to the increased permeability of the basal membrane of the renal glomerulus, which could be caused by different etiologies. It is more common among children; however, it can occur at any age [1]. Due to its distinct clinical and biological symptoms, relatively high incidence rate, and potentially related poor outcomes, it is important that medical students recognize it in clinical settings. Due to its physiopathology, nephrotic syndrome causes distinct and recognizable clinical and biological changes, such as peripheral edema, shortness of breath, proteinuria, and more, which could provide an interesting teaching opportunity to connect physiological, pathological, and clinical aspects with emphasis on history taking, clinical examination, differential diagnosis, and interpretation of laboratory results (eg, urinary sample).

In the medical faculty of the University of Geneva, Switzerland, a 2-hour session on teaching mentioned elements of nephrotic syndrome is typically held in a small group (10-12 students) and uses problem-based learning (PBL) based on a clinical vignette. Due to the ongoing COVID-19 pandemic, in-person teaching activities at our institution have been canceled. Videoconference-based lessons did not yield student engagement during the sessions. Therefore, our team sought to design an alternative learning activity to replace the traditional way of teaching nephrological semiology.

Electronic learning (e-learning) modules have been shown to be as effective as traditional teaching [2] and to improve novice learners’ performance in situated case-based learning [3]. Moreover, the use of rich media and visuals for teaching has been shown to improve and support learners’ experience, understanding, and engagement [4]. Finally, case-based and self-directed activities have been shown to be effective and to increase engagement and motivation for both students and teachers [5-7].

Redesigning our traditional activity as an e-learning module could therefore provide several advantages both in the context of the current pandemic as well as for future teaching activities. Students will have a didactic and validated source of information that meets the expectations of their educators. With the ubiquity of smartphones and other internet-connected devices [8-10], students will be able to easily access the module and repeat it as many times as they like, without being limited by location. Lastly, as this is a clinically pertinent subject, the module could serve as a reference later on in their clinical environment.

While several recent systematic reviews and meta-analyses provide evidence on the potentially beneficial nature of e-learning modules compared to traditional learning in terms of knowledge acquisition and clinical performance [11], important knowledge gaps exist on learners’ satisfaction with this type of teaching. Moreover, a paucity of evidence exists on the use and outcomes of e-learning interventions among undergraduate students and on the use of e-learning as a sole teaching method, especially in the context of clinical skills education.

In our paper, we aimed to prospectively study second-year (out of six) medical students’ satisfaction and perceptions of an e-learning module designed to replace the traditional teaching of nephrological semiology and compare the results to those of more advanced learners undertaking the same activity and evaluation. Our main hypotheses were that students would consider e-learning engaging due to the use of rich media and flexible since they would be able to perform it asynchronously, and that the proposed instructional design would be preferred to videoconference-based lessons.

## Methods

### Ethics Committee Approval

As the study was based on the analysis of anonymous data, no ethics committee approval from our institution was required.

### Clinical Skills Medical Curricula

In the medical faculty of the University of Geneva, Switzerland, clinical skills teaching begins in the second year of medical school and continues in a transversal fashion throughout the whole 6-year curricula [12]. Teaching is usually conducted in small groups, led by an experienced physician, and is reinforced by training sessions led by pretrained advanced medical students. Nephrological semiology is, as mentioned, one of the many clinical skills seminars offered by our faculty.

### Study Population

We prospectively included all second-year medical students (novice learners) from our institution. It should be noted that due to the COVID-19 pandemic, these students have never experienced in-person PBL classes. As data were anonymous, we do not have precise baseline characteristics; however, the students’ ages ranged from 20 to 27 years.

For comparison and to provide further insights, 17 fourth- to sixth-year medical students (advanced learners) were invited to undertake the same e-learning activity and evaluation. Of importance, all advanced learners previously participated in the same activity in its traditional in-person PBL form during their second year of medical school.

### e-Learning Instructional Design and Timing Considerations

The e-learning activity was designed using the latest evidence-based teaching methods. Instructional design was based on self-directed learning using a case-based learning activity of a patient presenting symptoms suspected of nephrotic syndrome. The learning objectives, which are listed in Table 1, were defined based on Bloom’s taxonomy for cognitive learning outcomes [13] and using the SMART (specific, measurable, achievable, relevant, and time-bound) criteria for well-defined objectives [14].

Students were provided with reading material to review before completing the e-learning module. The activity concentrated on history taking, clinical examination, differential diagnosis, laboratory examination, and treatment plans for a patient presenting with symptoms suspected of nephrotic syndrome.

The estimated time to complete both the readings and the e-learning module was approximately 2 to 3 hours long in a self-paced manner. Students were given 2 weeks to complete the module.

**Table 1:** Learning objectives for the nephrological semiology e-learning module within the cognitive learning domain (translated from French).

Number	Learning objectives
1	Cite the key body systems to concentrate on during system-based history taking of a patient suspected of nephrotic syndrome as evaluated by the self-assessment quiz.
2	List the key symptoms of nephrotic syndrome to look for during history taking as evaluated by the self-assessment quiz.
3	List the body systems to concentrate on during the clinical examination of a patient with a suspected case of nephrotic syndrome as evaluated by the self-assessment quiz.
4	List the differential diagnosis of peripheral edema as evaluated by the self-assessment quiz.
5	Cite the indicated paraclinical examination to be done in an ambulatory setting when suspecting nephrotic syndrome as evaluated by the self-assessment quiz.
6	Recognize the components of a urinary band strip typical of urinary infection, urinary lithiasis, and nephrotic syndrome by matching urinary elements to the corresponding syndrome in the evaluation quiz.
7	Cite the key feature of a urinary band strip for a patient with nephrotic syndrome as evaluated by the self-assessment quiz.
8	List the three elements of the diagnostic criteria of nephrotic syndrome as evaluated by the self-assessment quiz.

### Technology and Media Use

**Figure 1:**
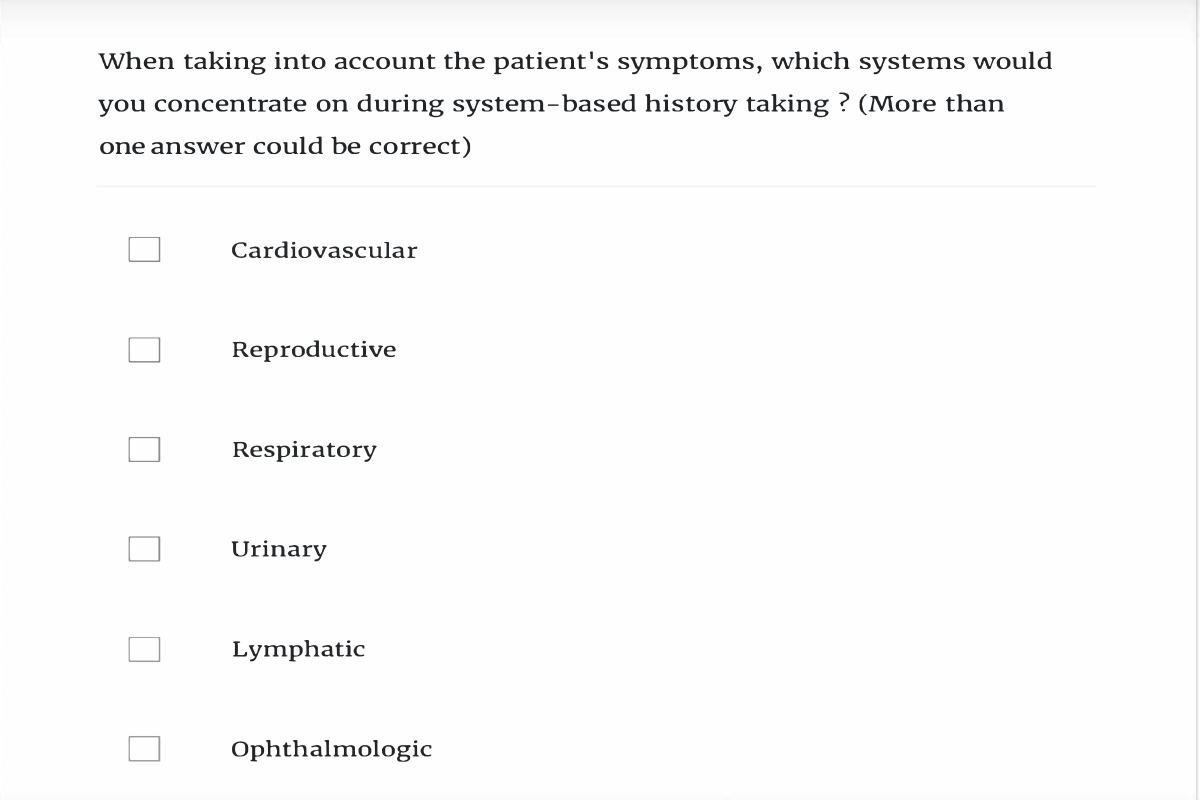
An example of a multiple-choice question from the e-learning activity.

**Figure 2:**
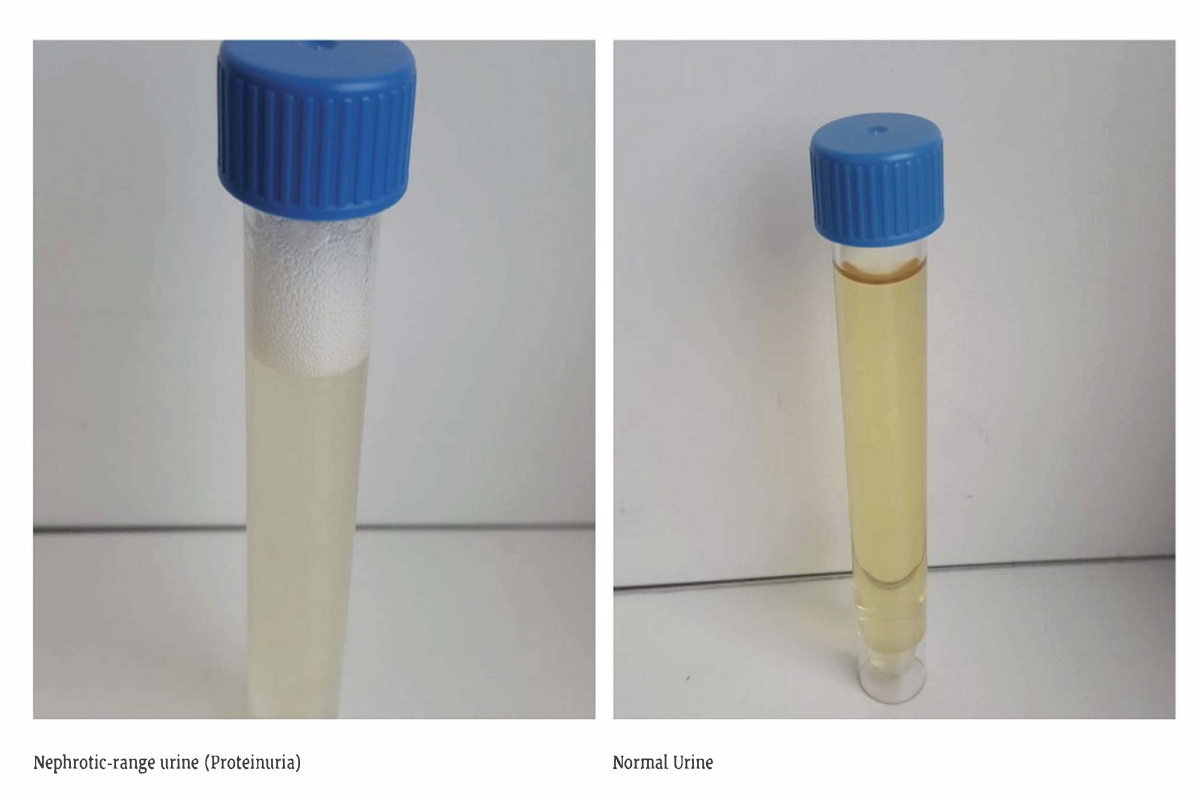
An example of the use of rich media in the e-learning activity.

**Figure 3:**
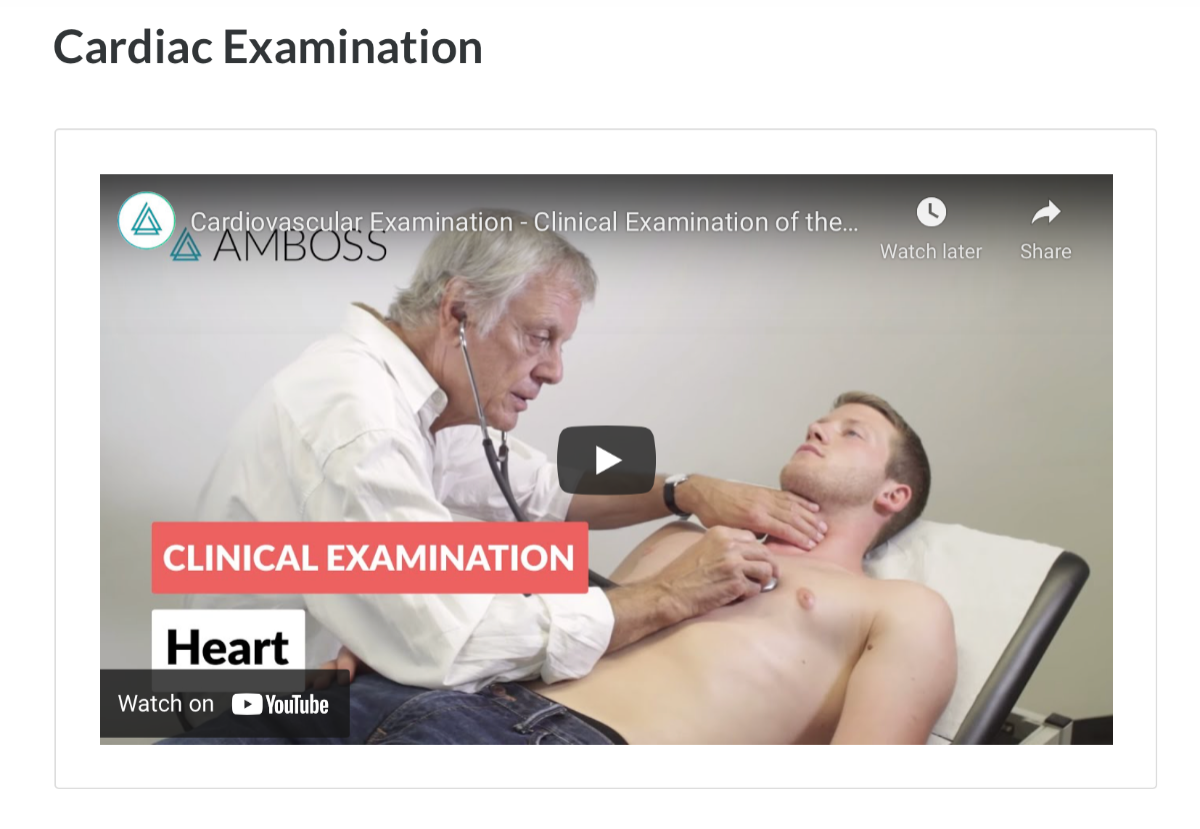
A locally validated and endorsed video of cardiac examination integrated into the e-learning activity [16].

**Figure 4:**
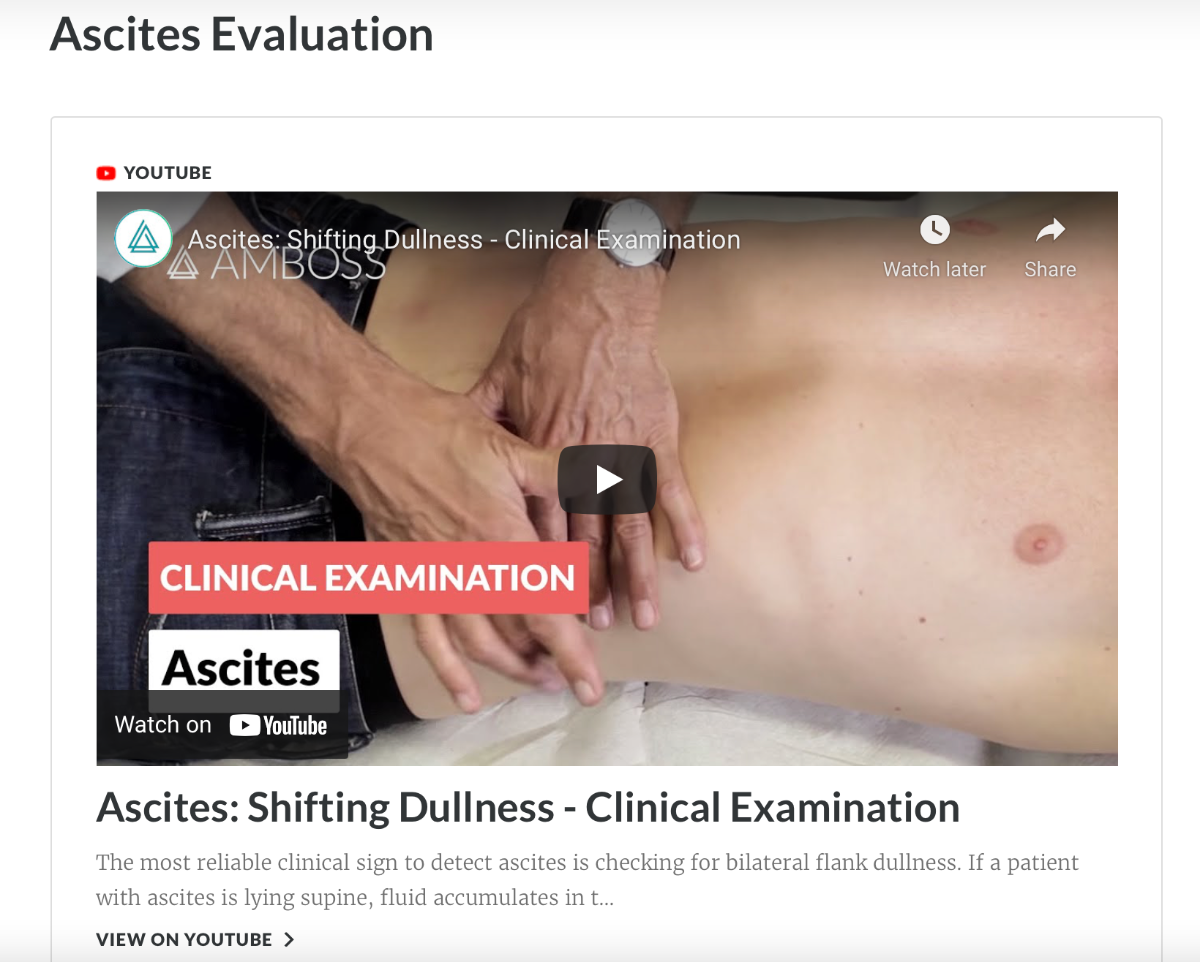
A locally validated and endorsed video of ascites evaluation integrated into the e-learning activity [16].

The e-learning module was created using Rise Articulate 360 (Articulate Global Inc) [15]. The activity itself was based on multiple-choice questions, text explanations, images, videos, and self-evaluation questions (Figures 1 and 2). The videos concentrated on cardiac, respiratory, and renal examination as well as skills to assess the presence of ascites. All videos were validated by expert faculty staff and were retrieved from the AMBOSS repository (Figures 3 and 4) [16].

### Activity Assessment

At the end of the module, students were provided with self-assessment multiple-choice questions based on knowledge acquired through the module.

### Postsession Evaluation

All students were given the opportunity to complete a 13-question survey. Survey questions were based on a validated tool (A Rubric for Evaluating E-Learning Tools in Higher Education) [17] and were conveyed using the SurveyMonkey platform (Momentive Global Inc) [18]. Students were asked to answer the questions using a 10-point Likert scale. A score of more than 7 for each question was considered a positive rating of the activity’s quality. In addition, a 1-hour videoconference session using Zoom (Zoom Video Communications) [19] was set in order to receive students’ live feedback and responses to questions regarding the learning activity.

### Statistical Analysis

A mixed methods analysis was performed. Quantitative data were presented as mean (SD). We compared data between the 2 student groups using the *t* test for means. Stata, version 16 (StataCorp LLC), was used for all statistical analysis [20]. A two-sided *P*<.05 was used to indicate significance. Qualitative data were analyzed using a thematic analysis approach.

## Results

### Overview

In total, 141 second-year medical students (novice learners) and 17 advanced medical students (advanced learners) undertook the e-learning activity. Among the novice learners, 88 (62%) students responded to the web-based survey, of whom 81 (92%) said they had completed the activity using their personal computer, 9 (10%) used a tablet, and 1 (1%) used a smartphone. Among advanced learners, all 17 students participated in the evaluation, of whom 12 (71%) used their personal computer and 5 (29%) used a smartphone to complete the activity.

### Survey Results

Both novice and advanced learners’ survey results are summarized in Table 2. Mean global satisfaction and user-friendliness of the e-learning activity was rated significantly higher among advanced versus novice learners (satisfaction: mean 8.7, SD 1.0 vs mean 7.3, SD 1.8, *P*<.001; user-friendliness: mean 9.0, SD 1.1 vs mean 7.6, SD 1.8, *P*<.001). Similarly, clarity and attainability of learning objectives were rated significantly higher among advanced versus novice learners (clarity: mean 9.6, SD 0.8 vs mean 7.7, SD 1.7, *P*<.001; attainability: mean 9.8, SD 0.5 vs mean 7.3, SD 1.3, *P*<.001). Both groups showed moderate to low preference for e-learning activities compared to videoconference-based activities, though e-learning preference was significantly higher among the advanced students (mean 7.5, SD 1.5 vs mean 5.8, SD 3; *P*=.02). Both groups showed a low and similar preference for in-person traditional PBL learning over the proposed e-learning module (mean 4.7, SD 2.7 vs mean 4.7, SD 1.8; *P*=.95). However, while significantly higher among advanced learners, both groups saw a high to extremely high need for the e-learning module to coexist and be complementary to traditional learning activities (mean 9.8, SD 0.5 vs mean 7.7, SD 2.5; *P*<.001). Both groups appreciated the integration of rich media in the e-learning module, with significantly higher satisfaction among the advanced learners (mean 9.5, SD 1.2 vs mean 8.2, SD 1.6; *P*<.001). Both groups highly and similarly appreciated the possibility to undertake the learning activity in an asynchronous fashion (mean 9.2, SD 1.2 vs mean 8.3, SD 1.9; *P*=.16) and moderately appreciated the lack of need to physically attend the class (mean 7.6, SD 2.5 vs mean 7.2, SD 2.6; *P*=.46).

**Table 2:** Comparison between novice and advanced learners’ web-based survey responses.

Question	Novice learners (n=88), mean (SD)	Advanced learners (n=17), mean (SD)	*P* value
Rate your overall satisfaction with the e-learning activity.	7.3 (1.8)	8.7 (1.0)	<.001
In my opinion, the interface used for the e-learning activity is user-friendly.	7.6 (1.8)	9.0 (1.1)	<.001
The learning objectives were clear to me.	7.7 (1.7)	9.6 (0.8)	<.001
I was able to attain the learning objectives thanks to the e-learning module.	7.3 (1.3)	9.8 (0.5)	<.001
I believe that the e-learning module is a more efficient way of teaching than videoconference-based (Zoom) activities (in terms of knowledge acquisition and in-class engagement).	5.8 (3)	7.5 (1.5)	.02
Compared to traditional learning (in-person problem-based learning classes), e-learning activities seem to me more efficient (in terms of knowledge acquisition and in-class engagement).	4.7 (2.7)	4.7 (1.8)	.95
This e-learning module needs to coexist as a complementary module to traditional teaching activities.	7.7 (2.5)	9.8 (0.5)	<.001
Integration of rich media (texts, quizzes, videos, images) facilitated my learning process.	8.2 (1.6)	9.5 (1.2)	<.001
The possibility to undertake the activity in an asynchronous fashion, according to my own time constraints, is a significant advantage for me.	8.3 (1.9)	9 (1.2)	.16
The possibility to undertake the activity via distance learning, without the need to physically attend the class, is a significant advantage for me.	7.2 (2.6)	7.8 (2.5)	.46

### Open-Ended Answers and Postactivity Zoom Session

A total of 22 (25%) novice learners and 11 (65%) advanced learners answered the open-ended comments section. Most comments from novice learners concentrated on three main themes. Students’ reflections are summarized in Tables 3 and 4. First, students stated that they enjoyed the activity in its present form; however, as formulized by 1 second-year student, “No activity can replace an in-person learning session with an experienced medical doctor.” Second, students from both groups suggested providing more text explanations for wrong answers to the multiple-choice questions. Finally, both novice and advanced learners showed interest in having the proposed e-learning module as a teaching activity complementary to in-person PBL classes for nephrological semiology.

During the postactivity Zoom session, the main themes discussed in the open-ended comments section were repeated by novice learners. Objective appreciation by the teachers leading the activity, however, indicated good attainability of the learning objectives, as manifested by the pertinent questions raised by students about the material and a good level of group discussion throughout the session.

**Table 3:** Novice learners’ qualitative inputs (translated from French).

Theme	Response
Advantages of blended learning	“The e-learning activity needs to co-exist with in-person learning, mainly because the in-person activity summarizes some points of the e-learning. Moreover, text-based material with the absence of a teacher makes memorization difficult.“ “Instead of the usual 2-hour Zoom sessions, it would be interesting to integrate the e-learning in the activity with 1-hour Zoom followed by the e-learning activity. This will help us integrate the information“ “I think that the e-learning activity is good but should be complementary to in-person activities.“ “This type of resource would be a great advantage for learning and to practice our skills. In fact, we often lack good references” “We should keep the e-learning as complementary to in-person activities. The in-person activity allows us to profit from the real-life and concrete experience of a physician”
Lack of explanations	“The e-learning activity presented answers to the questions but not enough explanations, which could have been more useful.” “Maybe the Q&A zoom session should be directly after the e-learning activity so we won’t forget our questions. Other than that, it was interesting and interactive, thank you” “I would have liked more explanations for the answers to the multiple-choice questions” “More explanations for the answers to the multiple-choice questions” “More explanations for the answers to the multiple-choice questions could help us better understand our errors”
Need for in-person group activities	“We already do everything by distance learning. I find it crucial that we could practice history taking with a real physician and be able to reflect together through group discussion. This will help me to better understand.” “The e-learning is well-made and stimulating but in-person PBL activity is primordial in my opinion” “Nothing can replace an in-person session” “The e-learning activity repeats the text in our references. An interaction with a real physician would be more stimulating, and hopefully more interesting than watching videos online. That said, the videos are useful for self-training” “Studying practical aspects with e-learning is difficult”

**Table 4:** Advanced learners’ qualitative inputs (translated from French).

Theme	Response
Advantages of blended learning	“Would be very useful to combine this activity with an in-person class by a nephrologist using more clinical vignettes and more teaching about nephrotic syndrome physiopathology. Nonetheless, the rich media is excellent and the interface very user-friendly”
Lack of explanations	“I feel that there are many elements of explanations that are lacking. In the in-person class, we had more detailed explanations, and it made it easier to understand” “Very good learning tool, maybe consider adding more photos for clinical signs as it will help us visualize and remember. Other than that, excellent learning method, which I would have loved to have earlier in the curricula” “Would be of value to add more explanations for wrong answers in the quizzes” “Very well-structured activity with a clear and logic sequence. Difficulty is adapted to the level of learners. Adding more explanations to wrong answers would be of value” “Write more explanations for wrong answers”
Need for in-person group activities	“Very good interface, fluid, and stimulating. I really appreciate the videos, and the quizzes allow a good synthesis of the learned material and help to achieve learning objectives. However, I am convinced that an in-person activity should be done in order to practice, and that the e-learning activity should exist in addition as it is very useful.”

## Discussion

### Principal Findings

Our study examined the satisfaction and perceptions of novice and advanced learners from an e-learning module designed to teach nephrological semiology as part of a mandatory clinical skills seminar targeted at second-year medical students in the medical faculty of the University of Geneva, Switzerland, during the COVID-19 pandemic. We provide several important insights. First, advanced learners showed significantly greater satisfaction with the e-learning activity and seemed to prefer this format over videoconference-based activities. Second, both novice and advanced learners seemed highly opposed to the idea of replacing in-person PBL activities with e-learning as the sole means of teaching but presented high interest in having the e-learning module as part of a blended learning approach. Finally, the integration of rich media seems to improve satisfaction and engagement for both novice and advanced learners in the presented e-learning module.

Several studies have already examined undergraduate students’ satisfaction and acceptability of e-learning–based activities [21,22] and have shown little or no significant preference for this type of learning. However, these studies were done outside of the current COVID-19 pandemic context, which presents a different learning reality where many novice learners never experienced in-person activities; this warrants the need to re-examine student satisfaction with and perception of traditional versus electronic-based activities. Only a few studies have examined this topic within the context of the current pandemic [23,24]; however, they considered videoconference-based activities as e-learning, which is different from the definition used in our study. Finally, major knowledge gaps exist concerning the acceptance and satisfaction of e-learning–based activities as the sole means of teaching compared to blended learning activities, especially for clinical skills undergraduate teaching, which in the current reality is of major importance in our opinion.

In fact, the current outbreak caused a major disruption within higher education and forced many institutions to revisit and reinvent teaching activities with the upscaling of online learning, as in-person teaching was not allowed for sanitary reasons [25]. Clinical skills teaching within health sciences education programs during pandemics presents a unique challenge for faculties as the need to teach and train competent future health care workers, who would soon actively participate in patient care, is put in the balance of their own safety as in-person teaching and contact with patients may come with the risk of infection. Therefore, finding and assessing alternative ways to teach clinical skills to future health professionals during this and future outbreaks is of significance.

In our study, novice learners showed only moderate satisfaction and attested to only moderate clarity and attainability of learning objectives after following the e-learning activity for nephrological semiology. Advanced learners, however, attested to a significantly higher level of satisfaction, as well as clarity and attainability of objectives, from the same activity. This could be explained in several ways. First, our instructional design was based on self-directed learning, which was shown to be more challenging and less accepted by beginners [26] as they might lack the maturity and experience to reach learning outcomes with minimally guided activities. This could explain as well why novice learners did not show a preference for the e-learning activity as compared to videoconference-based activities, even though students’ engagement with the latter was judged low by faculty members. Second, the novice learners in our study had never experienced in-person PBL lessons and seemed to be frustrated with distance-learning activities and feared not returning to in-person clinical skills activities as evidenced from the open-ended comments of the survey. This could have decreased their satisfaction with the activity, whereas advanced learners are more implicated in the clinical environment and have already experienced the same activity in its traditional form. Finally, as evidenced both by the survey responses as well as the postactivity videoconferencing session, certain novice students seemed to undertake the e-learning activity with their own learning agenda, which may not align with the actual learning objectives, a notion that has been demonstrated in past studies [27]. In fact, many of the comments from novice learners were related to their inability to practice renal and urological clinical examination, as these were not part of the activity’s learning objectives. This issue was further confirmed in the postactivity videoconference session, as objective evaluation by the teachers appraised good attainability of learning objectives.

Interestingly, both groups similarly agreed that online-based learning should not replace traditional in-person learning activities, and that blended learning, with the e-learning module as a complement to traditional teaching, could present an important learning benefit. This may be more evidence of medical students’ preference for in-person learning, especially in clinical skills education, as well as for the possible benefits of e-learning modules for life-long learning since this type of activity was highly valued by the students in our study who had already undertaken the activity in its traditional form and might have seen the module as an efficient repetition of already-acquired knowledge. Lastly, both student groups seemed to find asynchronous e-learning to be significantly advantageous in terms of individual time constraints, which could provide insights into the acceptability of this form of learning among medical students and as a means for increasing engagement in certain activities and earlier introduction of self-directed learning in the curricula.

### Strengths and Limitations

Our study has several limitations. The sample size of both groups was small, and the observational nature of this monocentric study could decrease the level of confidence in the results. Moreover, we did not directly compare traditional and online activities in different groups due to the pandemic situation. Finally, we did not assess knowledge retention using a standardized test as this was out of the scope of this study. However, the fact that both the activity and the evaluation were prospectively designed and were based on current evidence-based teaching methods and validated evaluation tools highlights important strengths of our work. The high survey response rate in both groups was another strength of the evaluation and may be indicative of undergraduate students’ motivation to actively participate in this curricular design. Lastly, the evaluation was made on an actual, ongoing teaching activity, which could provide real-world and important insights concerning learners’ satisfaction with e-learning–based activities.

### Conclusion

In the context of the current pandemic, novice medical students expressed only moderate satisfaction from an e-learning module intended as the sole means of nephrological semiology teaching and showed a clear preference for in-person, problem-based teaching complemented by e-learning for blended learning activities in the future. In addition, case-based e-learning activities might be better fitted for more advanced learners. Additional and larger studies should be performed to assess medical students’ satisfaction with online-based versus traditional learning activities in order to adapt the instructional design of alternative clinical skills teaching among health professionals and to improve preparation and clinical training in the context of future pandemics.

## Abbreviations

e-learning: electronic learning
PBL: problem-based learning
SMART: specific, measurable, achievable, relevant, and time-bound
